# Bilateral acute zonal occult outer retinopathy (AZOOR) in a young adult Indian male

**DOI:** 10.3205/oc000100

**Published:** 2019-04-04

**Authors:** Pukhraj Rishi, Ekta Rishi

**Affiliations:** 1 Shri Bhagwan Mahavir Vitreoretinal Services, Sankara Nethralaya, Chennai, India

**Keywords:** eye, acute zonal occult outer retinopathy, macula, maculopathy

## Abstract

A 31-year-old male presented with central scotoma of 9 months duration in the right eye and a similar complaint in the left eye, since a week. Best corrected visual acuity (BCVA) was 20/60 in the right eye and 20/30 in the left eye. Clinical features and supportive investigations were consistent with the diagnosis of acute zonal occult outer retinopathy (AZOOR). The patient was treated with systemic steroids. At 3-month follow-up visit, BCVA improved to 20/40 in the right eye and remained at 20/30 in the left eye. Humphrey’s visual field showed a slight reduction of scotoma in both eyes.

## Introduction

Acute zonal occult outer retinopathy (AZOOR) is an ocular syndrome characterised by an acute reduction in outer retinal function in one or more retinal zones, characterised initially by minimal fundus changes, enlarged blind spots, ERG abnormalities, and permanent visual field (VF) loss that is often associated with slow-progressing degeneration of the retinal pigment epithelial (RPE) cells [1], [2], [3]. It is mostly seen in young women and can be unilateral or bilateral. PubMed search revealed only one previous case report from India [4] and 4 relevant reports using keywords (AZOOR) (corticosteroids) [5], [6], [7], [8]. Hereby, we present a case of a 31-year-old Asian Indian male with bilateral presentation and treated with systemic steroids.

## Case description

**Figure 1:**
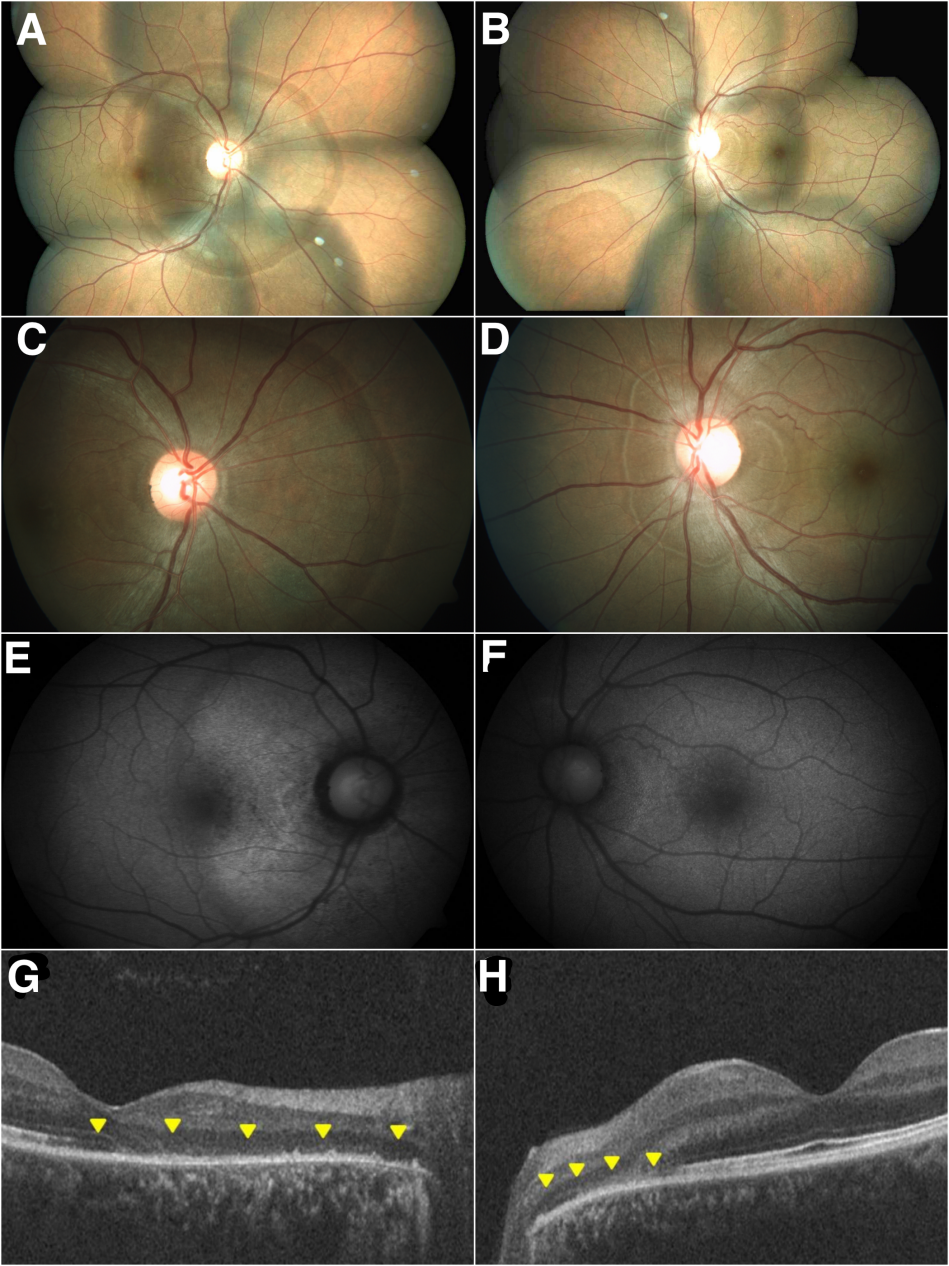
Colour fundus montage of the right (A) and left eye (B) shows a greyish opacification of the peripapillary retina; with more details of the posterior pole (C, D). FAF imaging reveals a speckled hyper-autofluorescence denoting an active disease phase and a speckled hypo-autofluorescence indicating RPE atrophy (E, F). OCT reveals a zonal loss of the outer retinal layers with a disruption of the photoreceptors (arrows; G, H).

A 31-year-old male presented with central scotoma of 9 months duration in the right eye and a similar complaint in the left eye, since a week. Best corrected visual acuity (BCVA) was 20/60 in the right eye and 20/30 in the left eye. His past history was unremarkable. Fundus examination revealed a circumscribed, flat, peripapillary, deep retinal lesion with a greyish, marginal opacification or demarcation line in both eyes; right larger than left (Figure 1 (Fig. 1)). 

**Figure 2:**
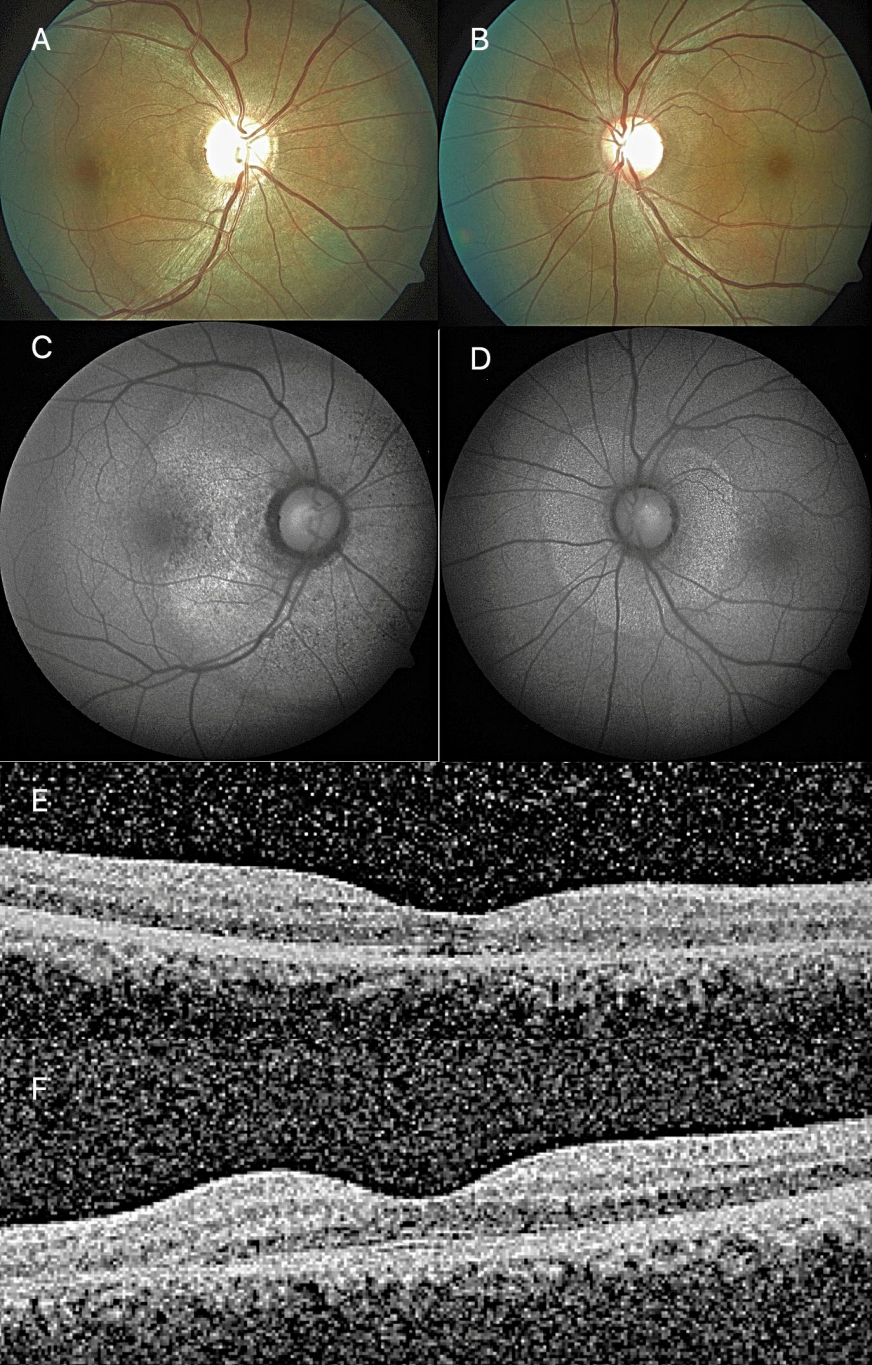
At 3-month follow-up, fundus reveals persistent AZOOR lesions with a marginal opacification in both eyes; right (A) larger than left (B). FAF imaging reveals a progressive hypo-autofluorescence at the margins (C, D). OCT scan of the right (E) and left (F) eye shows no change.

Fundus autofluorescence (FAF) imaging revealed a normal autofluorescence in the area beyond the demarcating line (zone 1), a speckled hyper-autofluorescence within the AZOOR lesion (zone 2), and a speckled hypo-autofluorescence corresponding to the development of choroidal atrophy (zone 3). The delineating line was predominantly hyper-autofluorescent in the left eye and hypo-autofluorescent in the right eye corresponding to the stage of the disease; hypo-autofluorescence corresponding to atrophic stage, hyper-autofluorescent corresponding to active stage (Figure 1 (Fig. 1)). Optical coherence tomography (OCT) was normal outside of the AZOOR line (zone 1). Inside the AZOOR line, multifocal material was present in the subretinal space resembling subretinal drusenoid deposits (zone 2). In zone 3, OCT showed a zonal loss of the outer retinal layers with a disruption of the photoreceptors, the ellipsoid line (formerly known as the inner and outer segment junction) and the interdigitation line (formerly known as cone outer segment tips, COST line) in both eyes with minimal sub-retinal fluid (SRF) in the left eye (Figure 1 (Fig. 1)). Humphrey’s visual field (HVF; 30-2) showed enlargement of blind spot in both eyes; right larger than left (Figure 2 (Fig. 2)). Multifocal electroretinogram (mfERG) showed a loss of foveal peak in the right eye and a reduced foveal peak in the left eye with normal parafoveal and perifoveal ring response in both eyes (Figure 1 (Fig. 1)). Clinical features and supportive investigations were consistent with the diagnosis of acute zonal occult outer retinopathy (AZOOR). After discussing the management options, treatment was initiated with oral steroids (1mg/kg body weight), tapered over 6 weeks. 

**Figure 3:**
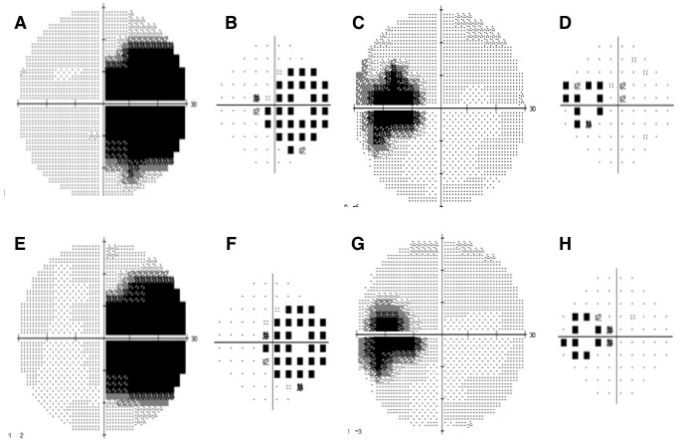
At baseline, HVF showing enlargement of blind spot with few temporal focal field defects in the right (A, B) and left eye (C, D). At 3-month follow-up, HVF shows a marginal reduction of scotoma in the right (E, F) and left (G, H) eye.

At 3-month follow-up visit, BCVA improved to 20/40 in the right eye and 20/30 in the left eye. Fundus examination revealed a circumscribed, flat, peripapillary, deep retinal lesion with a greyish, marginal opacification in both eyes; right larger than left (Figure 3 (Fig. 3)). Autofluorescence revealed the demarcation line assuming an incomplete or interrupted pattern with disease progression (Figure 2 (Fig. 2)). OCT showed a zonal loss of the outer retinal layers with a disruption of the photoreceptors, the ellipsoid and cone outer segment (COST) in both eyes (Figure 2 (Fig. 2)). Humphrey’s visual field (HVF; 30-2) showed a slight reduction of scotoma size in the left eye, and stable in the right eye (Figure 3 (Fig. 3)).

## Discussion

The diagnosis of AZOOR is deduced from the presence of a number of characteristic findings and ruling out other diseases. Usually, it is based on the course of symptoms, visual acuity changes, field defects, minimal fundus changes and corresponding visual fields, mfERG and OCT [2]. Our case had several features suggestive of AZOOR namely bilaterality, outer zonal affection of retina, presence of circumscribed lesions, and mild improvement with systemic steroids.

In summary, although AZOOR has been rarely reported from India, its possibility must be borne in mind in patients presenting with a bilateral visual impairment in the presence of circumscribed, flat, peripapillary, deep retinal lesions with a greyish demarcation line.

## Notes

### Competing interests

The authors declare that they have no competing interests.
